# The Effect of Diabetic Peripheral Neuropathy on Ground Reaction Forces during Straight Walking in Stroke Survivors

**DOI:** 10.1155/2017/5280146

**Published:** 2017-04-09

**Authors:** Amirah Mustapa, Maria Justine, Nadia Mohd Mustafah, Haidzir Manaf

**Affiliations:** ^1^Centre of Physiotherapy, Faculty of Health Sciences, Universiti Teknologi MARA, Puncak Alam Campus, 42300 Puncak Alam, Selangor, Malaysia; ^2^Discipline of Rehabilitation Medicine, Faculty of Medicine, Universiti Teknologi MARA, Sungai Buloh Campus, 47000 Sungai Buloh, Selangor, Malaysia; ^3^Clinical and Rehabilitation Exercise Research Group, Faculty of Health Sciences, Universiti Teknologi MARA, Puncak Alam Campus, 42300 Puncak Alam, Selangor, Malaysia

## Abstract

*Purpose*. The aim of this present study was to investigate the ground reaction forces (GRFs) alterations in stroke survivors with diabetic peripheral neuropathy (DPN).* Methods*. Ten stroke survivors with DPN, 10 stroke survivors without DPN, and 10 healthy controls with matched body weight between groups participated in this case-control cross-sectional study. Three-dimensional GRFs (anterior-posterior, medial-lateral, and vertical) were collected at a comfortable walking speed using the Nexus Vicon motion analysis system and force plate. The Kruskal–Wallis test was used to analyze GRFs parameters.* Results*. We found significant alterations of medial-lateral forces of the nonparetic side and vertical forces of the paretic side in stroke survivors with DPN compared to stroke survivors without DPN and healthy controls. In addition, there were smaller braking and lower propulsion peak in anterior-posterior forces, smaller magnitude of medial-lateral forces, and lower first and second peak of vertical forces in stroke survivors with DPN compared to stroke survivors without DPN and healthy controls.* Conclusion*. The study findings identified that GRFs were affected in stroke survivors with DPN on both the paretic and the nonparetic sides. Further investigations are warranted to explore the impact of DPN on the kinematics and muscle activity related to the gait performance in stroke survivors with DPN.

## 1. Introduction

Diabetic peripheral neuropathy (DPN) is a common debilitating microvascular complication of diabetics, particularly affecting 48.1 to 49.3% of individuals with both type 1 and 2 diabetes mellitus (DM) [1, 2]. DPN is characterized by a marked decrease of lower limbs sensation which causes delays in both proximal and distal peripheral sensory and motor neuron functions [3], consequently leading to musculoskeletal complications that are associated with biomechanical changes of movement [4] and deteriorations of gait performance [5]. In fact, these issues lead to high falls risk [6, 7].

It has been postulated that individuals with DPN exhibit significant deteriorations of gait performance compared to healthy controls or even persons with diabetes but no neuropathy symptoms [8]. A previous review also has concluded that DPN-related changes in the lower limbs may lead to gait performance variations, predominantly related to alterations of kinetic (such as reduced braking and propelling force), kinematic (such as increased hip flexion and knee extension), and spatiotemporal parameters (STP) (such as reduced gait speed and longer stance time) [9]. These alterations could be correlated to the adaptation of slower walking speed in DPN [10].

Kinetic parameters such as ground reaction forces (GRFs), joint powers, and moments are essential in the biomechanical aspect of gait due to their dependence on the kinematic and muscle activities [11]. Therefore, analysis of kinetic parameters, particularly GRFs, is compulsory in investigations regarding gait performance. It is already well known that DPN patients presented with alterations of GRFs during gait performance including reduce anterior-posterior forces production [12], lack of medial-lateral forces production, and lower peak of vertical forces [13]. These GRFs alterations are believed to develop as a result of progressive loss of somatosensory sensitivity, proprioception, and distal muscle function in DPN which cause decline of the GRFs production [13, 14] which might increase the instability of gait performance [14, 15].

While the relationship between DPN and GRFs alterations has been widely investigated, little is known about the GRFs production during gait performance in stroke survivors that have DPN. Few studies have demonstrated how GRFs are altered in the anterior-posterior, medial-lateral, and vertical axis during gait performance among poststroke survivors [16–18]. These alterations are associated with muscle weakness, motor incoordination, motor dysfunction, and impairments of balance in the poststroke phase [17, 18]. Motor dysfunctions in the poststroke phase are postulated to cause alterations of GRFs generating capacity which might result in instability of gait performance [16]. Hence, it is proposed that GRFs alterations in stroke survivors with additional DPN on the sound side can create destabilizing effects such as instability of gait performance.

In demand of a better understanding of the GRF alterations in gait performance among stroke survivors with DPN, an investigation of the GRFs production in stroke survivors with DPN is warranted. A comprehensive biomechanical investigation of stroke survivors with DPN may allow the identification of kinetic changes that adversely affect stability during gait. This knowledge may allow for a more precise formulation of tailored novel interventions to improve balance and stability in stroke survivors with DPN. Therefore, the aim of this present study was to investigate the GRFs production in stroke survivors with DPN compared to stroke survivors without DPN and healthy controls. We hypothesized that there was a significant alteration of GRFs generating capacity in the paretic and nonparetic side of stroke survivors with DPN compared to stroke survivors without DPN and healthy controls.

## 2. Methods

### 2.1. Participants Recruitment

This was a case-control cross-sectional study. Ten poststroke patients with DPN (9 males, 1 female), 10 poststroke patients without DPN (7 males, 3 females), and 10 healthy controls (3 males, 7 females) participated in this study. Stroke participants were recruited from a government funded hospital by using purposive sampling. Healthy controls were recruited from the local community. Comparative data was presented with body weight-matched groups. DPN was diagnosed based on a standardized clinical examination which included Semmes-Weinstein monofilament examination (SWME) and 128 Hz tuning fork [19]. The detection rate of DPN by using the 10 g SWME and 128 Hz tuning fork was nearly the same with a significant correlation between both tests [20]. In this study, the researcher applied SMWE from the medial site of the foot to the tibial site to diagnose DPN according to stocking-glove distribution in DPN [21]. Participants were included if they met the inclusion criteria: (1) at least 6 months after stroke, (2) able to obey 3-step command, and (3) able to walk 5 meters without aid. Participants were excluded if they have stroke at basal ganglia or cerebellum, other neurological conditions, severe musculoskeletal problems, other diseases that cause peripheral neuropathy other than type 2 DM, and foot ulcer. Participants were also excluded if they presented with visual field defects. The study received institutional ethics committee approval (600-IRMI (5/1/6)) and participants were given a written informed sheet prior to the participation.

### 2.2. Demographic Characteristics

Of the 10 participants recruited in stroke survivors with DPN group, 3 had hemorrhagic stroke and 7 had ischemic stroke, while 5 participants had hemorrhagic stroke and 5 had ischemic stroke in stroke survivors without DPN group. Stroke severity using the Stroke Rehabilitation Assessment of Movement (STREAM) [22] also was evaluated. The median STREAM score was 57.21 (range: 42.2–69) in stroke survivors with DPN and 66.79 (range: 52.5–69) in stroke survivors without DPN. For muscle strength evaluation, the median Motricity Index score was 44.5 (range: 27–75) on the paretic side and 63 (range: 57–99) on the nonparetic side in stroke survivors with DPN. The median Motricity Index score was 57 (range: 47–75) on the paretic side and 75 (range: 75–99) on the nonparetic side in stroke survivors without DPN.

### 2.3. Data Collection

A preliminary investigation including demographic measures was completed. The experiment was conducted in a gait lab that was equipped with Nexus Vicon motion analysis system (612 Oxford Metrics, Oxford, UK) with eight cameras (MX-F20) at a sampling rate of 100 Hz and Kistler force plates (type 28112A2-3S, Kistler Holding AG, Switzerland) mounted in the fixed platform. Anthropometry which includes leg length, knee width, and ankle width was measured and entered into the Plug-in-Gait (PiG) modelling software (Vicon, Oxford Metrics, Ltd.). Based on the PiG marker placement model, a total of 16 retroreflective spherical markers were placed bilaterally on the following anatomical landmarks: anterior superior iliac spines, posterior superior iliac spines, midthigh, lateral fibular head, mid calves, lateral malleolus, the base of the first metatarsal, and calcaneus [23]. Participants were asked to walk at a comfortable speed within 5 meters of the platform. Three practice trials were performed to familiarize the participants with the test before implementing 5 real trials with 5-minute rest between the tests to minimize fatigue effects.

### 2.4. Data Analysis and Statistical Tests

Raw marker data collected using the camera motion analysis system were filtered at 10 Hz using a low-pass fourth-order Butterworth filter. GRFs data were normalized by each participant's body weight. Three out of 5 recorded trials were chosen to determine the GRFs data with ensuring adequate foot contacts on the force plate. In some cases, multiple foot contacts on the force plate were averaged to get GRF values for each limb [24]. Paretic and nonparetic sides of stroke survivors were compared with a similar side of healthy control's limbs and the sides were categorized as paretic and nonparetic sides in the healthy controls group.

Nonparametric statistical tests were used to analyze the data by using the IBM SPSS statistical software version 20.0 (SPSS Inc.). Kruskal–Wallis test was used in measuring the GRFs data within three groups. *p* < 0.05 was considered statistically significant. When significant changes were observed, the Mann–Whitney *U* test with Bonferroni correction was performed to compare two clusters at a level of significance set at *p* < 0.016. Comparing GRFs data of the paretic and nonparetic side of each stroke group, further analysis by using Wilcoxon Signed Rank Test was used.

## 3. Results

Demographic information of participants is presented in Table 1. No significant age and body weight differences were found between groups. Among 10 participants in each group, 5 presented with left sided hemiparesis and 5 presented with right sided hemiparesis. The range of duration of the poststroke period was 8–60 months. GRFs data of paretic and nonparetic side between groups are shown in Table 2. Further analyses of comparing GRFs data of the paretic and nonparetic side of each stroke group by using Wilcoxon Signed Rank Test are shown in Table 3.

### 3.1. Anterior-Posterior Forces (GRFs Fy)

#### 3.1.1. Paretic Side

There were no significant differences in the magnitude of anterior-posterior forces of the paretic side between the three groups (*x*^2^(2) = 0.131, *p* = 0.937).

#### 3.1.2. Nonparetic Side

There were no significant differences in the magnitude of anterior-posterior forces of the nonparetic side between the three groups (*x*^2^(2) = 0.233, *p* = 0.890).

#### 3.1.3. Paretic and Nonparetic Side

Wilcoxon Signed Rank Test (Table 3), on comparing anterior-posterior forces of the paretic and nonparetic side of each stroke group, showed that there were no significant differences in stroke survivors with DPN (*p* = 0.494) and without DPN (*p* = 0.316), indicating that paretic and nonparetic sides in both stroke groups were fairly worse.

**Figure 1:**
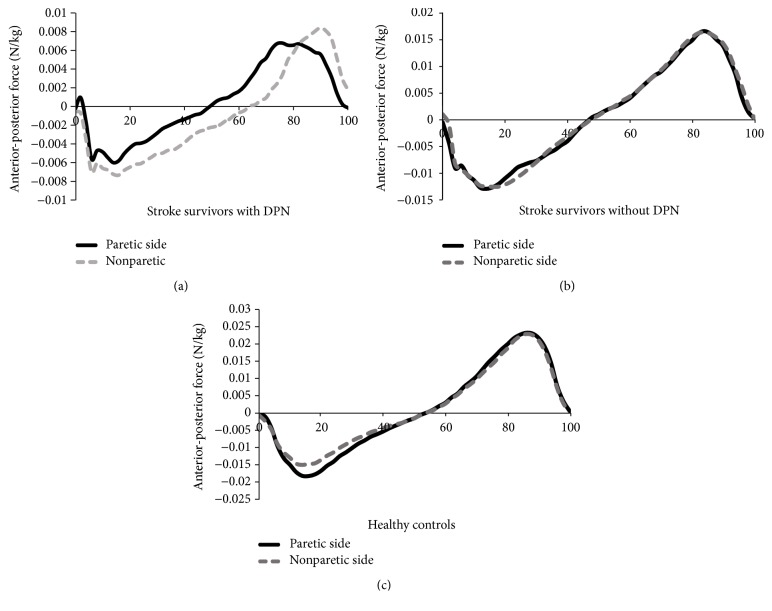
(a) Anterior-posterior forces of the paretic and nonparetic side of stroke survivors with DPN. (b) Anterior-posterior forces of the paretic and nonparetic side of stroke survivors without DPN. (c) Anterior-posterior forces of the paretic and nonparetic side of healthy controls. DPN: diabetic peripheral neuropathy.

Figure 1 shows the curves for anterior-posterior forces of the paretic and nonparetic side between the groups. Upon general visual inspection, anterior-posterior forces of the paretic and nonparetic side followed very similar patterns in stroke survivors without DPN and healthy controls. Meanwhile, the anterior-posterior forces of the paretic and nonparetic side of stroke survivors with DPN were quite different, with smaller braking forces in the first half and lower peak of propulsion forces in the second half of stance phase. The curve in the positive braking direction should roughly equal the curve in the negative propulsion direction to maintain steady-state walking speeds.

### 3.2. Medial-Lateral Forces (GRFs Fx)

#### 3.2.1. Paretic Side

There were significant differences in the magnitude of medial-lateral forces (GRFs Fx) of the paretic side between the three groups (*x*^2^(2) = 31.51, *p* = 0.001). A post hoc test showed that there were no significant differences of the paretic side between stroke survivors with and without DPN (*p* = 0.050). There were significant differences of the paretic side between stroke groups and healthy controls (*p* = 0.001).

#### 3.2.2. Nonparetic Side

There were significant differences in the magnitude of medial-lateral forces (GRFs Fx) of the nonparetic side between the three groups (*x*^2^(2) = 90.6, *p* = 0.001). However, there were significant differences of the nonparetic side between stroke survivors with and without DPN (*p* = 0.001). There were significant differences of the nonparetic side between stroke groups and healthy controls (*p* = 0.001).

#### 3.2.3. Paretic and Nonparetic Side

Wilcoxon Signed Rank Test (Table 3) showed that there were no significant differences in stroke survivors with DPN (*p* = 0.701) and significant differences in stroke survivors without DPN (*p* = 0.001). Therefore, this result indicated that the sound and impaired sides of lower limb's stroke were fairly worse in the presence of DPN in both limbs of stroke survivors with DPN compared to worsening of the impaired side in comparison to the normal side in stroke survivors without DPN.

**Figure 2:**
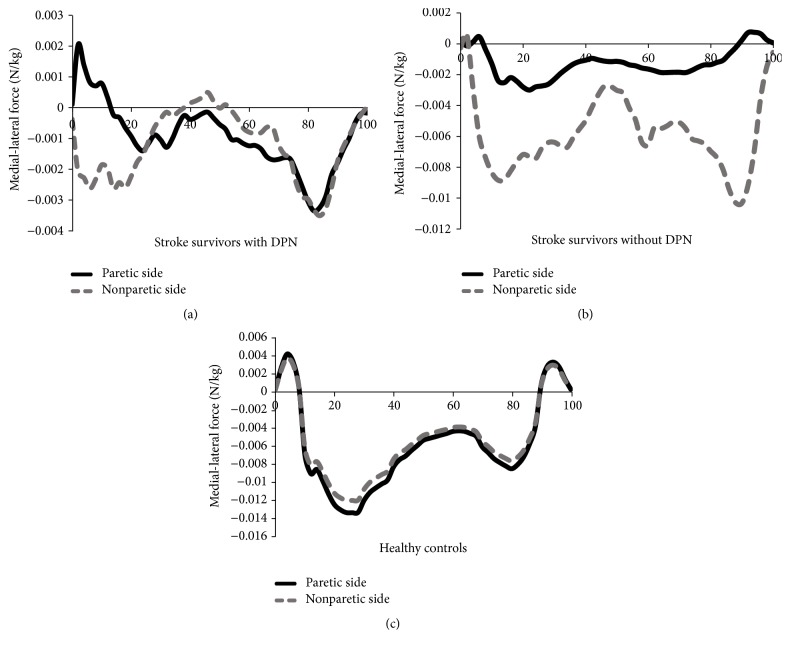
(a) Medial-lateral forces of the paretic and nonparetic side of stroke survivors with DPN. (b) Medial-lateral forces of the paretic and nonparetic side of stroke survivors without DPN. (c) Medial-lateral forces of the paretic and nonparetic side of healthy controls. DPN: diabetic peripheral neuropathy.

Figure 2 shows the curve for medial-lateral forces of the paretic and nonparetic side between the groups. Stroke survivors with DPN exhibited smaller force area, either the medial or the lateral force, compared to stroke survivors without DPN and healthy controls irrespective of the paretic and nonparetic side.

### 3.3. Vertical Forces (GRFs Fz)

#### 3.3.1. Paretic Side

There were significant differences in the magnitude of the vertical forces (GRFs Fz) of the paretic side between the three groups (*x*^2^(2) = 38.96, *p* = 0.001). A post hoc test showed that there were significant differences of the paretic side between stroke survivors with and without DPN (*p* = 0.001). There were significant differences of the paretic side between stroke groups and healthy controls (*p* = 0.001).

#### 3.3.2. Nonparetic Side

There were significant differences in the magnitude of the vertical forces (GRFs Fz) of the nonparetic side between the three groups (*x*^2^(2) = 20.74, *p* = 0.001). However, there were no significant differences of the nonparetic side between stroke survivors with and without DPN (*p* = 0.545). There were significant differences of the nonparetic side between stroke groups and healthy controls (*p* = 0.001).

#### 3.3.3. Paretic and Nonparetic Side

Wilcoxon Signed Rank Test (Table 3) showed that there were significant differences in stroke survivors with DPN (*p* = 0.001) and in stroke survivors without DPN (*p* = 0.014), indicating that the paretic side was worse than the nonparetic side in both stroke groups.

**Figure 3:**
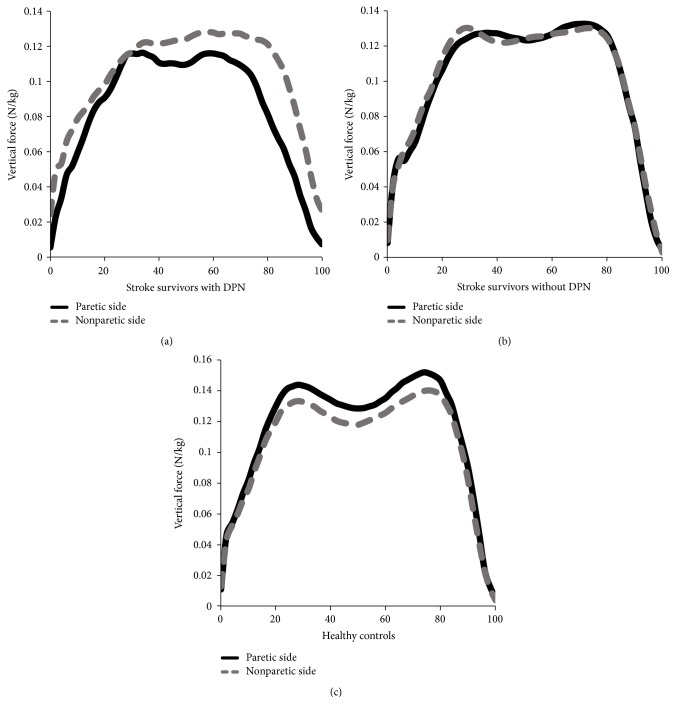
(a) Vertical forces of the paretic and nonparetic side of stroke survivors with DPN. (b) Vertical forces of the paretic and nonparetic side of stroke survivors without DPN. (c) Vertical forces of the paretic and nonparetic side of healthy controls. DPN: diabetic peripheral neuropathy.

Figure 3 shows the curve for vertical forces of the paretic and nonparetic side between the groups. Upon general visual inspection, stroke survivors with DPN did not demonstrate the classic double bump throughout that stance phase for both paretic and nonparetic sides compared to other groups. While following similar paths to healthy controls, stroke survivors without DPN showed lower magnitude of vertical force in the paretic side than in the nonparetic side.

## 4. Discussion

The aim of this study was to reveal the impacts of DPN on GRFs in the stroke survivors. We found significant alterations of medial-lateral forces of the nonparetic and vertical forces of the paretic side in stroke survivors with DPN compared to stroke survivors without DPN and healthy controls. We also found a general nonsignificant trend toward alterations of anterior-posterior forces on both the paretic and the nonparetic sides, medial-lateral forces on the paretic side, and vertical forces on the nonparetic side in stroke survivors with DPN. This is probably because of the presence of DPN on both the sound and the impaired sides which cause impaired force generating capacity during gait performance.

Impaired force generating capacity in DPN is possibly due to the following reasons. The first reason is the motor control deteriorations following progressive loss of somatosensory sensitivity, impaired proprioception, and distal muscle weakness in the lower limbs following DPN [25]. The second reason is the input information alteration from the lower limbs due to the loss of tactile sensitivity, especially in vastus lateralis (VL) muscles [25]. In addition, delayed activation of these VL muscles might cause a deficiency in the mechanism of shock absorption at the initial heel strike phase [26] with a sequent of decreased vertical forces production [27]. Plantar sensitivity of the foot following DPN also influences GRFs alterations. Additionally, compensation offered by the nonparetic limb through the phenomenon of “impairment and compensation” proposes that the greater the impairment of the paretic limb, the greater the compensation offered by the nonparetic limb to maintain normal kinetic pattern during gait performance [12]. Therefore, the deteriorations of GRFs exhibited in this study by the paretic and the nonparetic side were equivalent in the presence of DPN in both limbs.

Upon general visual inspection, anterior-posterior forces of both the paretic and the nonparetic sides of stroke survivors with DPN were quite different compared to the other groups, with smaller braking forces in the first half and lower peak of propulsion forces in the second half of stance phase. This finding is similar to the investigation of DPN and stroke group which found smaller braking and propulsive forces in the DPN and stroke groups compared to the controls group [12, 28]. Smaller braking and propulsion impulse indicated decreased push-off ability in the body's preparation for the swing phase [29] and also occurred in manifestations of adopting a slower walking speed [13].

Another interesting observation in this study was that stroke survivors demonstrated lower forces in the medial direction of the paretic and the nonparetic side compared to healthy controls, which is probably because of the preparation of foot abduction movement for the toe-off phase. This finding opposed the finding by Scott-Pandorf et al. [29] who found that the peripheral arterial disease population displayed higher forces in the medial direction than in the lateral direction. Higher forces in the medial direction indicated increased step width with decreased single support time and longer double support time for the purpose of maintaining wider stance during gait [29]. The opposite of these findings might be explained by the different types of participants' diseases. For example, Scott-Pandorf et al. [29] studied the kinetic pattern in the peripheral arterial disease population, while the stroke population in this present study presented lack of wider stance in hemiplegic gait developed since their stroke. In addition, prolonged positive and negative lateral forces in the paretic and the nonparetic side of stroke survivors with DPN at the initial stance phase could be interpreted as lack of foot adduction in the heel contact with the purpose of larger width stance to improve gait stability [29, 30].

Stroke survivors with DPN did not demonstrate the classic double bump throughout the stance phase of vertical forces for the paretic and the nonparetic side compared to other groups. The pattern of asymmetry for vertical forces observed in this study has also been seen by others [17, 31]. The flatter configuration might be produced due to the absence of normally deep mid stance as a result of longer double support time and an inability to fully extend the limb at the single support phase [29]. While stroke survivors without DPN and healthy controls followed similar paths, vertical forces peak of the paretic side tended to be lower in magnitude than on the nonparetic side. In contrast, the first and second peaks of vertical forces were higher in the DPN when compared with the control and diabetic groups [14, 30]. This overload might be associated with changes of the intrinsic musculature of the foot in DPN that undergo higher compressive and prolonged longitudinal stresses during walking [14, 27]. This discrepancy in our findings might be related to the manifestations of adopting slower walking speed in stroke survivors with DPN which then leads to lower vertical forces production.

Therefore, stroke survivors with DPN may have further GRFs alterations as their lower limbs generated lower forces, regardless of the paretic or the nonparetic side due to the impairments of balance, weakness, motor incoordination, and motor dysfunction [16, 28]. We noted several limitations in this study. First, the present results were obtained from a relatively small sample size, which probably cannot be generalized to individuals with stroke and DPN. Second, the cross force plate's position mounted in the platform might cause difficulty for participants to have adequate foot contact on the force plate. Thus, participants might adopt a variable walking pattern during data collection which might affect the GRFs production. Lastly, we have relied solely on clinical measures (SWME and turning fork) combination with medical record to identify and categorize stroke survivors with DPN. Thus, we recommended determination of DPN using vibration perception threshold (VPT) < 25 volts [32] in the future studies to get valid identification of stroke survivors with DPN.

## 5. Conclusion

In summary, this present study showed that stroke survivors with DPN had pronounced alterations in GRFs compared with stroke survivors without DPN and healthy controls. These interpretations were consistent with our hypotheses. To the best of our knowledge, this might be the first study that investigated the impacts of DPN on the GRFs in the stroke survivors. Therefore, it attained the knowledge of the impacts of DPN on the kinetic patterns in the stroke survivors. This present study also suggested the importance of gait training among the stroke survivors with DPN, which would reduce the number of fall incidences. Besides that, training of the sensory and motor functions such as muscle strength is highly recommended for the stroke survivors with DPN to obtain optimal gait patterns after stroke. Further investigations are warranted to explore the effects of DPN on the kinematic and muscle activity that can be related to the gait performance in chronic stroke survivors.

## Figures and Tables

**Table 1:** Demographic information of participants.

Characteristics	Stroke survivors with DPN (*n* = 10)		Stroke survivors without DPN (*n* = 10)		Healthy controls (*n* = 10)		*p* value
	Median	Range	Median	Range	Median	Range	
Age (years)	58	50–65	54.5	41–65	50	41–61	0.051
Weight (kg)	70.5	60.9–103	72.1	64.4–88.65	67.35	50.5–82	0.223
Height (cm)	161.1	156–180	162.1	152.8–170	154.2	141.6–174	0.033^*∗*^
Hemiparesis side (left/right)	5/5	—	5/5	—	—	—	—
Duration of stroke (months)	23.5	8–60	28.5	8–60	—	—	0.594

DPN: diabetic peripheral neuropathy.

^*∗*^Significant.

**Table 2:** GRFs of the paretic and nonparetic side.

GRFs	Stroke survivors with DPN (*n* = 10)		Stroke survivors without DPN (*n* = 10)		Healthy controls (*n* = 10)		*p* value	Post hoc (*p* value)		
	Median	Range	Median	Range	Median	Range		SDPN versus S	SDPN versus HC	S versus HC
Anterior-posterior forces (paretic) (N/kg)	−0.0002	−0.01–0.01	0.0008	−0.01–0.02	−0.0006	−0.02–0.02	0.937	—	—	—
Anterior-posterior forces (nonparetic) (N/kg)	−0.0009	−0.01–0.01	0.0008	−0.01–0.02	−0.0008	−0.02–0.02	0.890	—	—	—
Medial-lateral forces (paretic) (N/kg)	−0.001	0.00-0.00	−0.0014	0.00-0.00	−0.0024	0.00-0.00	0.001^*∗*^	0.050	0.001^*∗*^	0.001^*∗*^
Medial-lateral forces (nonparetic) (N/kg)	−0.009	0.00-0.00	−0.0003	0.00-0.00	0.02	−0.04–0.11	0.001^*∗*^	0.001^*∗*^	0.001^*∗*^	0.001^*∗*^
Vertical forces (paretic) (N/kg)	0.09	0.00–0.11	0.12	0.00–0.13	0.13	0.00–0.15	0.001^*∗*^	0.001^*∗*^	0.001^*∗*^	0.001^*∗*^
Vertical forces (nonparetic) (N/kg)	0.12	0.02–0.13	0.12	0.00–0.13	0.13	0.01–0.15	0.001^*∗*^	0.545	0.001^*∗*^	0.001^*∗*^

DPN: diabetic peripheral neuropathy.

GRFs: ground reaction forces.

SDPN: stroke survivors with DPN.

S: stroke survivors without DPN.

HC: healthy controls.

^*∗*^Significant.

**Table 3:** Comparison of GRFs of the paretic and nonparetic side.

GRFs	Stroke survivors with DPN (*n* = 10)	Stroke survivors without DPN (*n* = 10)
	(*p* value)	(*p* value)
Anterior-posterior forces (N/kg)	0.494	0.316
Medial-lateral forces (N/kg)	0.701	0.001^*∗*^
Vertical forces (N/kg)	0.001^*∗*^	0.014^*∗*^

DPN: diabetic peripheral neuropathy.

^*∗*^Significant.

## References

[1] Katulanda P., Ranasinghe P., Jayawardena R., Constantine G. R., Sheriff M. H. R., Matthews D. R. (2012). The prevalence, patterns and predictors of diabetic peripheral neuropathy in a developing country. *Diabetology and Metabolic Syndrome*.

[2] Kiani J., Moghimbeigi A., Azizkhani H., Kosarifard S. (2013). The prevalence and associated risk factors of peripheral diabetic neuropathy in Hamedan, Iran. *Archives of Iranian Medicine*.

[3] Dixit S., Maiya A. (2014). Diabetic peripheral neuropathy and its evaluation in a clinical scenario: a review. *Journal of Postgraduate Medicine*.

[4] Gupta A., Gupta Y. (2014). Diabetic neuropathy: Part 1. *Journal of the Pakistan Medical Association*.

[5] Rani P., Raman R., Rachapalli S., Pal S., Kulothungan V., Sharma T. (2010). Prevalence and risk factors for severity of diabetic neuropathy in type 2 diabetes mellitus. *Indian Journal of Medical Sciences*.

[6] Schwartz A. V., Vittinghoff E., Sellmeyer D. E. (2008). Diabetes-related complications, glycemic control, and falls in older adults. *Diabetes Care*.

[7] Wallace C., Reiber G. E., LeMaster J. (2002). Incidence of falls, risk factors for falls, and fall-related fractures in individuals with diabetes and a prior foot ulcer. *Diabetes Care*.

[8] Paul L., Ellis B. M., Leese G. P., McFadyen A. K., McMurray B. (2009). The effect of a cognitive or motor task on gait parameters of diabetic patients, with and without neuropathy. *Diabetic Medicine*.

[9] Fernando M., Crowther R., Lazzarini P. (2013). Biomechanical characteristics of peripheral diabetic neuropathy: a systematic review and meta-analysis of findings from the gait cycle, muscle activity and dynamic barefoot plantar pressure. *Clinical Biomechanics*.

[10] Katoulis E. C., Ebdon-Parry M., Lanshammar H., Vileikyte L., Kulkarni J., Boulton A. J. M. (1997). Gait abnormalities in diabetic neuropathy. *Diabetes Care*.

[11] Raja B., Neptune R. R., Kautz S. A. (2012). Gait: relation to kinetic and kinematic parameters. *Journal of Rehabilitation Research & Development*.

[12] Savelberg H. H. C. M., Schaper N. C., Willems P. J. B., De Lange T. L. H., Meijer K. (2009). Redistribution of joint moments is associated with changed plantar pressure in diabetic polyneuropathy. *BMC Musculoskeletal Disorders*.

[13] Raspovic A. (2013). Gait characteristics of people with diabetes-related peripheral neuropathy, with and without a history of ulceration. *Gait and Posture*.

[14] Saura V., dos Santos A. L. G., Ortiz R. T., Parisi M. C., Fernandes T. D., Nery M. (2010). Predictive factors of gait in neuropathic and non-neurophatic diabetic patients. *Acta Ortopedica Brasileira*.

[15] Sawacha Z., Spolaor F., Guarneri G. (2012). Abnormal muscle activation during gait in diabetes patients with and without neuropathy. *Gait and Posture*.

[16] Wang J., Hurt C. P., Capo-Lugo C. E., Brown D. A. (2015). Characteristics of horizontal force generation for individuals post-stroke walking against progressive resistive forces. *Clinical Biomechanics*.

[17] Neckel N. D., Nichols D., Hidler J. M. Joint moments exhibited by chronic stroke subjects while walking with a prescribed physiological gait pattern.

[18] Kim B. J., Robinson C. J. (2005). Postural control and detection of slip/fall initiation in the elderly population. *Ergonomics*.

[19] Menz H. B., Lord S. R., St George R., Fitzpatrick R. C. (2004). Walking stability and sensorimotor function in older people with diabetic peripheral neuropathy. *Archives of Physical Medicine and Rehabilitation*.

[20] Al-Geffari M. (2012). Comparison of different screening tests for diagnosis of diabetic peripheral neuropathy in primary health care setting. *International Journal of Health Sciences*.

[21] Tanenberg R. (2009). Diabetic peripheral neuropathy: painful or painless. *Hospital Physician*.

[22] Ahmed S., Mayo N. E., Higgins J., Salbach N. M., Finch L., Wood-Dauphinée S. L. (2003). The Stroke Rehabilitation Assessment of Movement (STREAM): a comparison with other measures used to evaluate effects of stroke and rehabilitation. *Physical Therapy*.

[23] Manor B., Wolenski P., Li L. (2008). Faster walking speeds increase local instability among people with peripheral neuropathy. *Journal of Biomechanics*.

[24] Bowden M. G., Balasubramanian C. K., Neptune R. R., Kautz S. A. (2006). Anterior-posterior ground reaction forces as a measure of paretic leg contribution in hemiparetic walking. *Stroke*.

[25] Akashi P. M. H., Sacco I. C. N., Watari R., Hennig E. (2008). The effect of diabetic neuropathy and previous foot ulceration in EMG and ground reaction forces during gait. *Clinical Biomechanics*.

[26] Sacco I. C. N., Amadio A. C. (2000). A study of biomechanical parameters in gait analysis and sensitive cronaxie of diabetic neuropathic patients. *Clinical Biomechanics*.

[27] Giacomozzi C., Caselli A., Macellari V., Giurato L., Lardieri L., Uccioli L. (2002). Walking strategy in diabetic patients with peripheral neuropathy. *Diabetes Care*.

[28] Sharma S., McMorland A. J. C., Stinear J. W. (2015). Stance limb ground reaction forces in high functioning stroke and healthy subjects during gait initiation. *Clinical Biomechanics*.

[29] Scott-Pandorf M. M., Stergiou N., Johanning J. M., Robinson L., Lynch T. G., Pipinos I. I. (2007). Peripheral arterial disease affects ground reaction forces during walking. *Journal of Vascular Surgery*.

[30] Giacomozzi C., D'Ambrogi E., Uccioli L., MacEllari V. (2005). Does the thickening of Achilles tendon and plantar fascia contribute to the alteration of diabetic foot loading?. *Clinical Biomechanics*.

[31] Lewek M. D., Bradley C. E., Wutzke C. J., Zinder S. M. (2014). The relationship between spatiotemporal gait asymmetry and balance in individuals with chronic stroke. *Journal of Applied Biomechanics*.

[32] Roman De Mettelinge T., Delbaere K., Calders P., Gysel T., Van Den Noortgate N., Cambier D. (2013). The impact of peripheral neuropathy and cognitive decrements on gait in older adults with type 2 diabetes mellitus. *Archives of Physical Medicine and Rehabilitation*.

